# Not To Be Forgotten: Jaroslav Madlafousek’s Important Contributions to Sex Research

**DOI:** 10.1007/s10508-025-03218-y

**Published:** 2025-08-18

**Authors:** James G. Pfaus, Marek Špinka

**Affiliations:** 1 Department of Psychology and Life Sciences, Faculty of Humanities, Charles University, Pátkova 2137/5, Praha 8 – Libeň, 18200 Prague, Czech Republic; 2 Center for Sexual Health and Interventions, Czech National Institute of Mental Health, Klecany, Czech Republic; 3 Department of Ethology and Companion Animal Science, Faculty of Agrobiology, Food and Natural Resources, Czech University of Life Sciences, Prague, Suchdol, Czech Republic; 4 Department of Psychology, Faculty of Arts, Charles University, Prague, Czech Republic

**Keywords:** Czech, Sexology, Humans, Rats, Plethysmography, Paraphilias

## Abstract

Jaroslav Madlafousek, Ph.D., C.Sc. (1922–2008) was a Czech comparative psychologist, physiologist, ethologist, and sexologist. He made extremely important empirical contributions to the study of sexual behavior in both humans and rats, among others, helping to pioneer the use of plethysmography to measure genital sexual arousal in men. Here, we present a biography of his life, his struggles against the dictatorships that ruled his country, and his scientific and clinical achievements, many of which were decades ahead of their time but still remain unknown to researchers outside Czechia. We include a complete bibliography of his scientific contributions in both Czech and English journals in the supplementary material.

**Supplementary Information:**

The online version contains supplementary material available at 10.1007/s10508-025-03218-y.

## Introduction

**Fig. 1:**
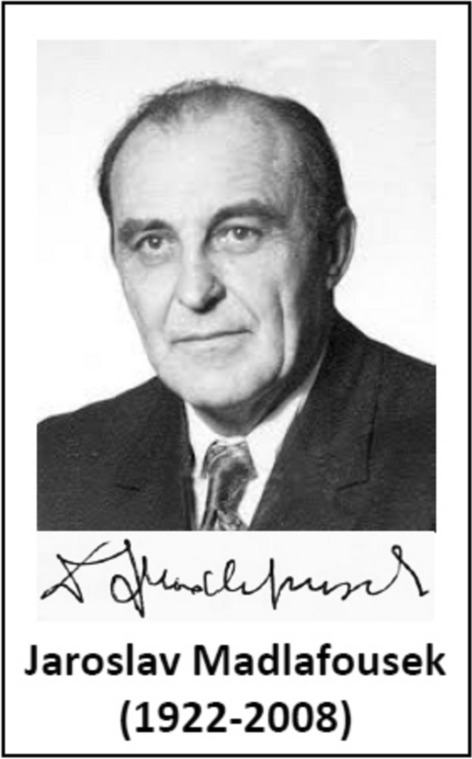
Jaroslav Madlafousek

As with other scientific disciplines, our understanding of sexual behavior is built upon the work of those that came before us. Unfortunately, some of that work and the people who did it can be lost to history. The pioneering research of Jaroslav Madlafousek (Fig. 1) is a good example.

Madlafousek was a postwar Czech comparative psychologist, ethologist, physiologist, and sexologist, and his research on motivation in general, and both human and rat sexual behavior in particular, was far ahead of its time. He was one of the most original, creative, but underappreciated researchers and thinkers regarding the evolutionary roots of human mind and behavior. In the 1950s, he developed a version of impedance plethysmography and, in the 1960s, utilized both impedance and volumetric plethysmography for use in studies of human male sexual arousal and helped Czech psychiatrist Kurt Freund apply the latter as a diagnostic tool for problematic paraphilias. He was one of three founders of the Psychiatric Research Institute at the Prague Psychiatric Center in Bohnice in 1960–1961. (The other two were Kurt Freund and Lubomír Hanzlíček, who became its first director.) He also founded the study of ethology in the former Czechoslovakia, and he applied ethological approaches to the study of sexual motivation and courtship in both humans and rats. In the late 1960s, he and his student, zoologist Zdeněk Hliňák, decoded the intricacies of appetitive sexual behaviors displayed by female rats as part of their control of sexual interaction with males, and delineated the role of steroid hormone priming of those behaviors. He studied the effects of sexual experience in the transition from appetitive to consummatory sexual behavior in male rats, and the role of the preoptic area (POA), steroid hormones, and serotonergic drugs in the control of these phases of sexual behavior in both rats and Arctic foxes. He applied the knowledge obtained from his animal studies to humans, notably examining the nonverbal behavior of opposite-sex pairs in natural conditions and comparing it afterward to their subjective assessment of the attractiveness of the other partner. He examined the role of preparatory erotic stimulation in exhibitionists, pedophiles, and men with violent sexual fantasies. He published his findings in both Czech and English journals, including three papers in the *Archives of Sexual Behavior* (see supplementary file for his full bibliography and additional photographs).

## Youth

Madlafousek was born on 9 May 1922 in Jičín, a town 95 km northeast of Prague. His father was a high school teacher and local cultural worker (someone who plans, organizes, and manages events like concerts, theater shows, exhibitions, etc.). His mother was a homemaker. He had a sister. He had musical training as a violinist (something he continued to love throughout his life) and enjoyed sports and nature. But, above all, he was engaged intellectually and enjoyed learning about science.

## Second World War and the Postwar Period

The German Army invaded Czechoslovakia in October 1938 to take the Sudetenland, and on 15 March 1939, two months shy of Madlafousek’s 17th birthday, occupied all of Bohemia and Moravia. Madlafousek finished his gymnasium (high school), but had to suspend his entry to university as Czech universities were closed by the Nazis in November 1939. During the Nazi occupation, the so-called Protectorate of Bohemia and Moravia was controlled by several high-ranking German officials, including Reinhard Heydrich, one of the organizers of the *Kristallnacht*, founder of the notorious SS Einsatzgruppen, and chief architect of the Final Solution (Shoah). As Czech culture was suppressed under the Nazis and people deported to camps or forced into Nazi war production, Madlafousek joined the Czech resistance network known as ÚVOD (Ústřední vedení odboje domácího). Not much is known about his experiences in the resistance during World War II, but he managed to escape the mass murder of Czech people suspected of aiding the Czech paratroopers that assassinated Heydrich in late May 1942 as part of Operation Anthropoid. He participated in the Prague uprising against the Nazis on 5–9 May 1945, just before the arrival of the Soviet Red Army. He later stated that he had worked as an advisor for the Vocational Counseling Center in Jičín from July 1942 to the end of June 1945.

Madlafousek was 23 when the war came to an end. As universities reopened, he promptly enrolled in the Department of Psychology at the Faculty of Arts at Charles University in Prague (Filozofická fakulta Univerzity Karlovy v Praze*,* or FF UK) where he became interested in motivation. He was mentored by Josef Stavěl, one of the founders of Czech psychology, and studied the behavioral rules that governed funnel sandtrap construction by antlion hill larvae (*Myrmeleon formicarius*). After completing his undergraduate studies, Stavěl took him on as a graduate student and exposed him to work by Tinbergen and Lorenz. He and Stavěl had many debates over the relative value of instinct versus learning in the control of motivation. Madlafousek was heavily influenced by Tinbergen because of his layered concept of motivational states, a concept that Madlafousek continued to develop for decades to come. He rejected the idea that motivation had to be controlled exclusively by drive and instinct, and instead supported the notion that experience with external factors such as rewards, punishments, contexts, and different incentive releasing stimuli could alter motivation. He found Lorenz’ hydraulic analogy of motivation too simplistic and drive oriented. Madlafousek also liked to tinker with devices and testing apparatus to come up with more ecologically valid experimental testing environments and the right detection tools. During his graduate studies, he formed a close intellectual relationship with another student of Stavěl, future physiologist and ethologist, Josef Lát (1918–1988), with whom he would collaborate throughout his life, especially in the areas of hypothesis testing and statistics. During his undergraduate and graduate training, Madlafousek was employed as a research assistant at the Czech Institute of Human Labor in Prague from October 1948 to the end of June 1951, where he worked with Lát and an eclectic mix of psychologists, physiologists, hygienists, technicians, lawyers, and pedagogues, an experience that reinforced his belief in the power of interdisciplinary teams that could debate alternative ideas. He became dedicated to free, independent, and multidisciplinary thinking, and brought that to bear in a rigorous scientific way on both experimental questions and social problems. As a concrete example, Madlafousek maintained that it is always better—and for real progress in a particular problem even necessary—to examine and assess two or more on-par hypotheses at time, rather than just test dichotomously a single hypothesis against its default (and boring) null hypothesis.

## The Communist Takeover, Graduation, and His Early Work

**Fig. 2:**
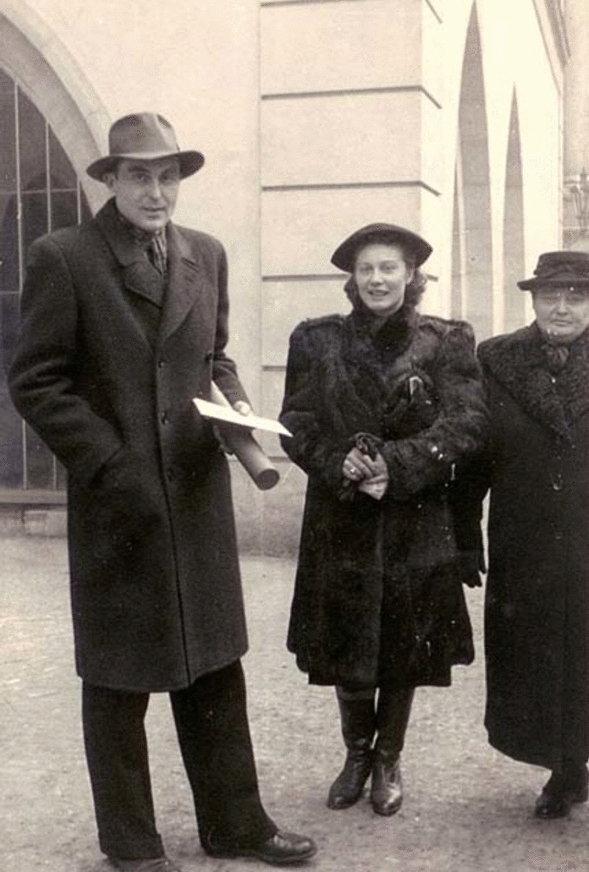
Madlafousek in the fall of 1949 after his Ph.D. graduation (with his sister and mother)

**Fig. 3:**
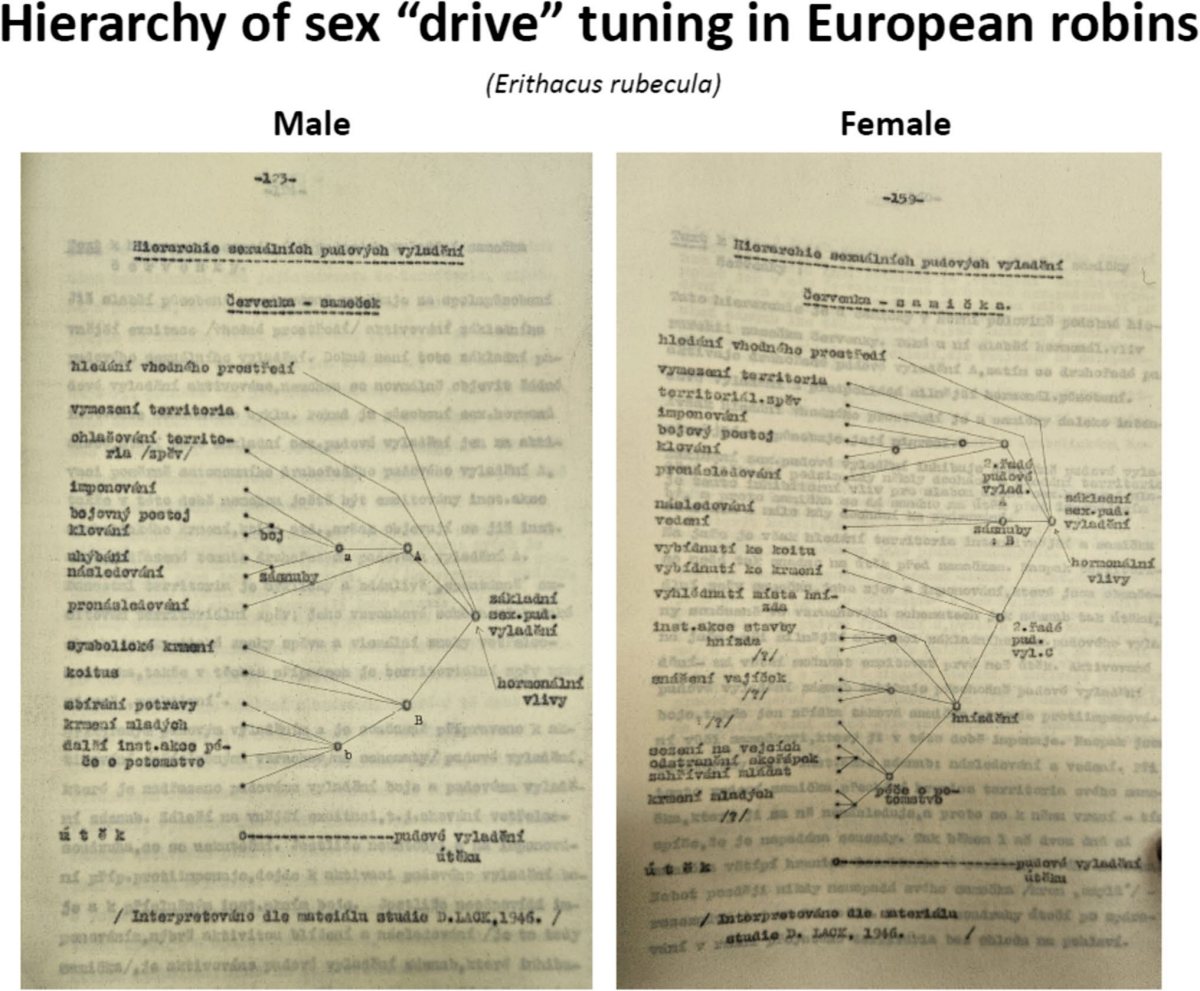
Two pages from Madlafousek’s thesis that contrast ethograms of sexual behavior (left side) displayed by male and female European robins as a function of hormonal influences, instinct (“fixed action patterns”), and engagement with the environment (context, partner behavior, clutch size). His analysis of these behaviors came from the published work of British evolutionary ecologist David Lack (1910–1973). His thesis used this kind of ethogram to contrast the sexual behavior of male and female lizards, fish, and birds. This allowed a truly comparative analysis of the relative hormonal, instinctual, and environmental conditions that influenced sexual motivation. He wrote that such an analysis could be applied to humans, something he did later to reveal releasing stimuli for people with problematic alcohol use or paraphilic behaviors

**Fig. 4:**
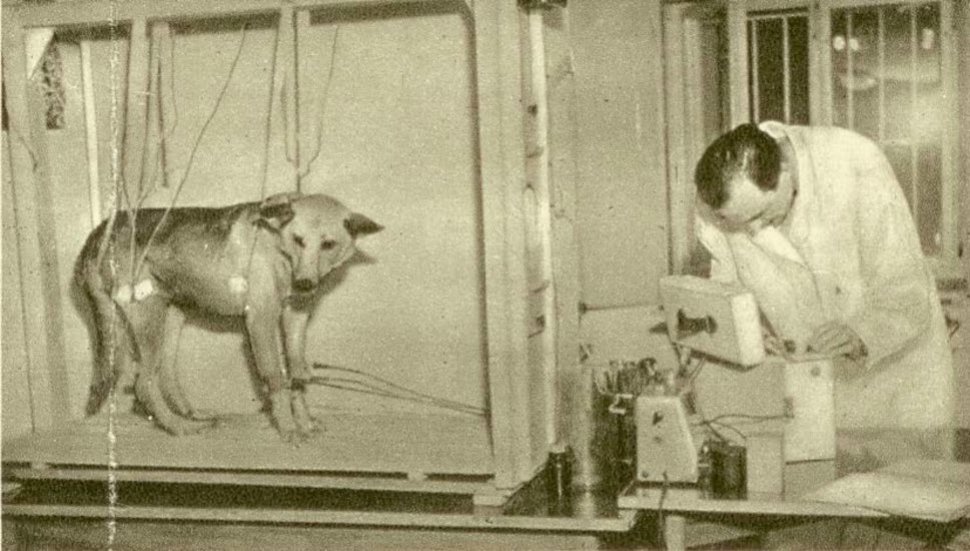
Madlafousek in the 1950s at the Institute of Blood Circulation

In the parliamentary elections of 1946, the Communist Party of Czechoslovakia (KSČ) won a plurality and entered into a coalition government. Their leader, Klement Gottwald, became Prime Minister. By 1947, however, the popularity of the KSČ had declined. Fearing defeat in the elections of 1948, the KSČ, already under full Soviet control, took control of the government in late February through an armed coup and, a few months later, completed full state control with Gottwald assuming the presidency. The Communist Party would thus rule Czechoslovakia for the next 41 years. It was under this backdrop that Madlafousek completed his Ph.D. in late 1949 (Fig. 2) with his 206-page thesis entitled “Contributions to the psychology of animal motivation” (Fig. 3). From 1951 to 1954, he was an assistant professor in the Department of Psychology at FF UK, where he gave a course on comparative psychology. He also met and married his partner, Vlasta Gyringerová. From July 1951 to March 1952, he worked as a psychologist at the First Children’s Clinic in Prague. He also worked at the newly established Institute for Cardiovascular Disease Research (Ústav pro choroby oběhu krevního, or ÚCHOK, in Czech) in Prague-Krč from 1951 to 1956, where he developed impedance plethysmography in dogs (Fig. 4), and later for humans (adapting and modifying the “andrograph” of Czech sexologist Josef Hynie), to analyze blood accumulation in different tissues, including the penis (e.g., Fencl et al., 1959; Gerova & Madlafousek, 1956). During this time, he was prodded to join the Communist Party, but refused. In fact, he became a well-known critic of the Communist regime, and this got him into repeated trouble. For example, several months following the Hungarian uprising (October–November 1956), Madlafousek was dismissed abruptly from his job at the Institute, presumably for his political beliefs, as were others following “obedience checks” during the so-called Purge of the Intelligentsia (1957–1958). After being out of work for a few months, his situation reached the attention of Jaroslav Skála, the director of the Anti-Alcohol Treatment Center in Lojovice, 31 km SW of Prague, who promptly hired him as a researcher. It was here that Madlafousek began to use an ethological approach to behavior analysis, as he had done in his Ph.D. thesis, to categorize the motivation of alcoholics to drink, and especially those who relapse to drinking. He noted that certain releasing stimuli, e.g., having a clique of friends that were drinkers, bottles of alcohol, bars, other alcohol-related cues, smoking, and even general stressors, initiated craving. Dealing with those cues, then, became the focus of a new therapeutic approach that countered the use of more common aversion techniques (Madlafousek, 1961). He also discovered Sidney Siegel’s 1956 book on nonparametric statistics, and utilized it for many future data analyses. His research here did not go unnoticed, especially by Kurt Freund.

## Psychiatric Research Institute at Bohnice: Phallometry and Rat Sex

Psychiatric research flourished in the late 1950s and early 1960s, aided by the application of new pharmacological and behavioral treatments for psychiatric disorders like depression and schizophrenia. In 1960, Madlafousek, Freund, and psychiatrist Lubomír Hanzlíček established the Psychiatric Research Institute at the Prague Psychiatric Center in Bohnice, a few km north of Prague’s old city center. It was here that Madlafousek established his rat laboratory in the basement of Pavilion 19 and began his ethological studies of rat sexual behavior, most notably on the role of steroid hormones in appetitive and consummatory aspects of female sexual behavior (work he did with Zdeněk Hliňák, who defended his thesis in 1974), how those behaviors altered the appetitive and consummatory sexual behaviors of male rats, and how brain regions like the POA were involved. Freund collaborated with him on those studies, but also on more well-known experiments using volumetric penile plethysmography (PPG, or what Freund termed “phallometry”) to the study the reaction of males with problematic paraphilias to stimuli that elicited their sexual arousal (also with sexologist Aleš Kolářský). In the mid- to late 1950s, Freund utilized PPG as a diagnostic tool for homosexuality (Freund, 1957; Freund et al., 1965) and became evident that homosexuality was an inborn trait that could not be changed. Freund and Madlafousek utilized PPG to screen out men faking homosexuality to get out of compulsory two-year military service, which was unpopular. However, the method was never used by them to identify homosexual men to the police or for discrimination, as homosexual acts were decriminalized in Czechoslovakia in 1961 under the provisions of the New Criminal Procedure Act of 1961 (Zákony pro lidi, 2025). During the 1960s, Madlafousek and Freund developed both volumetric and impedance plethysmography as tools for experimental psychopathology in humans. During this time, Madlafousek and his student Hliňák began publishing their rat work in Czech and English journals and in 1968 Madlafousek was elected Chair of the Czechoslovak Psychological Society for a two-year term. He had begun to receive national and international recognition of his research findings, and he was invited in 1968 to attend and present his findings at the XI International Ethological Conference in Rennes, France, scheduled for 1969.

## “Prague Spring” and the Aftermath

The year 1968 was pivotal in Czechoslovakia. In early January, the new First Secretary of the KSČ, Alexander Dubček, initiated reforms, including dividing the country into a federation of separate Czech and Slovak states, partial decentralization of the economy, limiting the power of the secret police, democratization, and a loosening of restrictions on the media, speech, and travel. This “Prague Spring” upset the Soviets, who invaded the country on August 21, 1968, along with other Warsaw Pact troops to suppress the reforms and oust Dubček. In the year following, some Czech scientists fled the country, including Freund, who went to Toronto, Canada and established his phallometry laboratory at the Clarke Institute of Psychiatry, now the Centre for Addiction and Mental Health. Freund collaborated with many past and current sex researchers there, including Ray Blanchard, Ron Langevin, Robin Wilson, and Ken Zucker. Many others, including Madlafousek, chose to stay. During the subsequent period of “Normalization” (Czech: normalizace), Gustáv Husák replaced Dubček and reversed all reforms including those for foreign travel. However, Madlafousek (along with other Czech ethologists and psychologists, like Drs. Věra Nováková, Jaroslav Šterc, and Vitězslav Bičík) had already received state approval to attend the XI International Ethological Conference, so a special exception was made. This was to be Madlafousek’s only foreign conference, but it allowed him to establish links with other ethologists and psychologists from the West, particularly from North America. He had been permitted previously to travel to Poland (1962) and the Soviet Union (1963) to meet with ethological colleagues there.

Despite his growing national and international reputation in comparative psychology and ethology, Madlafousek remained only an occasional lecturer in the Department of Psychology at FF UK. However, at the Psychiatric Research Institute in Bohnice, his rat sexual behavior research with Hliňák, PPG work with Kolářský, and nonverbal communication work on human courtship with psychologist Michael Žantovský (who would later become the Czech ambassador to the US, UK, and Israel) began taking off (see supplemental materials). Another of Madlafousek’s former students, Luděk Bartoš, was founding a Department of Ethology at the Animal Production Research Institute (now the Institute of Animal Science) in Uhříněves, 20 km SW of the Psychiatric Research Institute in Bohnice. With the support of the Institute’s director, Bartoš persuaded Madlafousek to join them, where he stayed as a faculty member until his retirement in the late 1990s. In 1973, he co-organized the first yearly Czechoslovak Ethological Conference. He also focused his attention on the role of the POA in male copulatory behavior (Madlafousek et al., 1970), and on the appetitive motivational aspects of sexual interaction (e.g., Hliňák & Madlafousek, 1968; Madlafousek & Hliňák, 1970, 1977). His observations regarding appetitive sexual behaviors in female rats were among the first accounts of females being “active” in their control of the male. He created ethograms of the dyadic sexual behavior of rats that showed clearly how female solicitations not only compelled males to chase them, but also altered the strength of the male’s intromissions and the latency to ejaculate (e.g., Hliňák & Madlafousek, 1972; Madlafousek & Hliňák, 1977; Madlafousek et al., 1976). During this time, Madlafousek was visited by colleagues from around the world. In one instance, he was visited by an American who wanted to repay him for his hospitality. The colleague arranged for Madlafousek to receive an invitation for a longer study stay abroad, financed by research funds from the North Atlantic Treaty Organization (NATO). At that time of the Cold War, however, NATO was officially regarded as an “imperialist military group” and the de facto “enemy” of the Soviet Union and Warsaw Pact nations. Thankfully, Madlafousek managed to catch this invitation in time and cover it up. This “imperialist” invitation alone could have meant an absolute end to his scientific career.

**Fig. 5:**
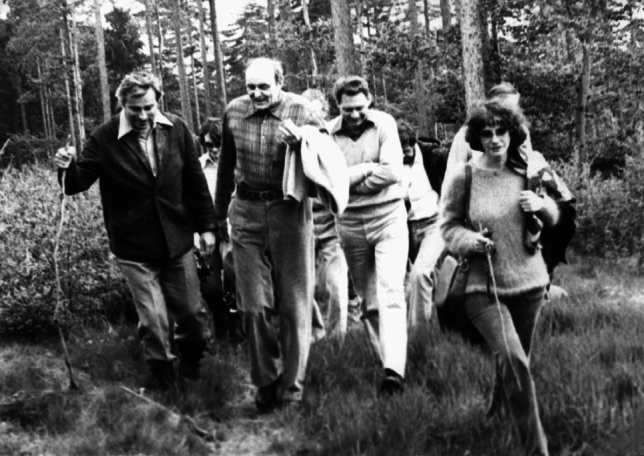
Madlafousek leading a field hike during the Czechoslovak Ethological Conference in Zvíkov in 1982 (age 60)

Madlafousek’s most productive period, from the standpoint of research and publications, was from the 1960s to the early 1990s. His publications in both English and Czech caught the eye of other ethologists, behavioral neuroendocrinologists, pharmacologists, and sexologists studying rat sexual behavior, and in particular, the famous behavioral neuroendocrinologist Frank Beach (President of the International Academy of Sex Research from 1976–1977). Beach cited a number of findings from Madlafousek’s laboratory in his seminal paper on sexual attractivity, proceptivity, and receptivity in female mammals (Beach, 1976). Madlafousek and Hliňák’s careful observations about the inventory, patterning, and measurement of sexual behavior in the female rat were published the following year (Madlafousek & Hliňák, 1977), and the analysis of female appetitive precopulatory behaviors was presented as an ethogram along with the relative stimulation of those behaviors by estradiol and progesterone, and an analysis of what kind of behavior they stimulated in the male. This preceded the critical analyses of female rat sexual behavior by McClintock and Adler (1978), McClintock (1984) and Erskine (1985), which, when taken together, led to the creation of both unilevel and bilevel pacing chambers (Erskine, 1985; Mendelson & Gorzalka, 1987; Pfaus et al., 1990, 1999), and to the findings of Paredes and colleagues regarding the role of paced copulation in female sexual reward (Paredes & Alonso, 1997; Paredes & Vazquez, 1999). In turn, appetitive female sexual behaviors became important preclinical indices for sexual desire in women (e.g., Ågmo & Laan, 2022; Pfaus et al., 2003, 2023), especially when they predicted the efficacy of treatments that increased desire, like the anti-serotonergic drug flibanserin or the melanocortin agonist bremelanotide, along with the effect of sexual nonreward in dampening desire (Pfaus, 2009; Pfaus et al., 2012; Quintana et al., 2022). His work in human males during this time refined the use of PPG as a diagnostic tool to examine physiological sexual arousal in men with problematic paraphilias (e.g., Kolářský & Madlafousek, 1980, 1983). Here, he used his concept that the course of sexual behavior is governed by a set of motivational states that adaptively follow one another, and that the cause of some aberrant male sexual behaviors may reside in an atypical ordering of these states in some individuals. This conceptualization echoed Ellis (1933) and added a motivational state analysis to the disrupted courtship patterning proposed by Freund and Kolářský (1965). He extended this to problematic paraphilias as a means of determining therapeutic efficacy, stressing that affected males can more easily control and channel their sexual behavior into societally acceptable forms if they understand the motivational causes of their urges. As a committed Darwinist, he believed strongly that behavior and mental states were determined by evolution, but that the capacity to learn in species’-typical ways was an evolutionary trait that allowed individuals to understand their world by particular experiences of it, which of course gave rise to diversity in behavioral responses to external stimuli. He believed that such experiences were age- and state-specific, linking his thinking squarely to Life History Theory. Many of his students and colleagues recounted with joy the scientific retreats he would hold at his cottage in Orlík nad Vltavou, 80 km south of Prague, full of data and spirited debates. Likewise, he would organize and lead much beloved nature hikes for attendees at the Czech and Slovak Ethological meetings (Fig. 5).

## The “Velvet Revolution” and After

The events of 1989 in Eastern Bloc countries saw both violent and peaceful transitions from Communist dictatorships to democracy. In Czechoslovakia, this occurred from 16 to 28 November 1989, when students and faculty from Charles University, along with many dissidents, demonstrated against the government. As more and more people occupied Wenceslaus Square in Prague, and elsewhere in the country, the Communist Party simply caved in, relinquished power, and resigned. This was called “The Velvet Revolution.” In his last official act, Husák appointed the first largely non-Communist government since 1948. Dissident poet and playwright Václav Havel became president, and Alexander Dubček was elected speaker of the new Federal Parliament. In December of 1992, the Czech and Slovak regions split into two separate countries that subsequently entered the European Union. Prior to this, however, Madlafousek had laid the groundwork to continue yearly meetings of a unified Czech and Slovak Ethological Society. He attended the 1992 meeting of the International Academy of Sex Research in Prague (organized by Jan Raboch and Jaroslav Zvěřina) and provided a report of the meeting for the official bulletin of the Czech and Slovak Ethological Society a couple of months later. He continued to write and attend meetings after his retirement and hold occasional retreats for former students and colleagues at his cottage. He became concerned about children and their upbringing, and how to teach them to become happy and empathic adults. He complained that we have sexology and gynecology, but no “parentology,” and he worried that ad hoc educational campaigns based on Euro-American theories of sociocultural behavioral shaping did more harm than good. He started writing a book about the evolution of child care and the intuition of parents to nurture talent, happiness, and confidence in their children. He never finished it. He died of heart failure on 27 June 2008 at the age of 86.

## Glimpses of Madlafousek the Person

The glimpses below were pieced together from various recollections made by people who knew Madlafousek personally. It is striking that no one had any negative comments. Nearly all remarked how his steadfast and stubborn resistance to Soviet-era Communism likely kept him from developing his career further, but how it led Czech scientists to respect him all the more.

Madlafousek liked to be called by his nickname “Jarka.” In addition to his native Czech, he was fluent in German, Russian, French, and English. He was tall, almost 200 cm (6′5″), and fit, keenly playing volleyball with much younger colleagues in the garden of the Psychiatric Institute well into his 60s. He was gregarious and outgoing. He and his wife Vlasta referred to each other as *beruška* and *beruško* (Czech for “ladybug”). He has been described as a wonderful human being, honest, principled, and uncompromising, yet kind and understanding. He hated flattery and hypocrisy. He was a great mind, and he appreciated that in others. He loved his work and being part of a team. He enjoyed hiking and fishing from a rowboat that he carefully repaired. He did not suffer fools well, especially party apparatchiks that tried to politicize science and scientists that were eager to please them. He maintained his independence and dignity as a person and scientist. He did not care about fame as a scientist; but rather the creation and dissemination of real knowledge. He was rigorous with his experimental designs and analyses. As a committed Darwinist, he believed in the power of the ethological approach to studying behavior. He liked good-natured arguments. Words truly mattered to him, and he maintained that he was first and foremost a psychologist. It took years for colleagues like Bartoš to almost convince him to consider himself an ethologist as well. He was a proud member of the Čapek Brothers’ Society. He was so serious about providing accurate descriptions of behavior that he would mimic the zig-zag dance of stickleback fish, solicitations (including ear-wiggling!) and lordosis postures of female rats, and the mounting behavior of male rats, much to the delight of his students and colleagues. His lectures were described as captivating and insightful.

There are not many photographs of him smiling. He rarely laughed but his eyes always expressed an inviting, friendly, and deep interest in the person he was talking to. One wonders what his eyes saw between 1939 and 1945. One wonders how he must have felt trying to continue his research while others were trying to pull the rug out from under his career. He believed strongly in the great prospects and utility of behavioral sciences, but was deeply troubled by his too-frequent encounters with what he considered bad science. One wonders what might have happened had he been allowed to travel freely to international meetings in the prime of his productivity. The former students that we interviewed, now older persons themselves, say that he inspired them to go into research or clinical work, and that he set a high and rigorous standard for the quality of that work. His research with Freund helped establish PPG as a research and diagnostic tool. His findings anticipated the study of incentive-based motivation, of nonverbal courtship behavior, of the importance of female sexual motivation, the regulation of sexual motivation and behavior by hormones and experience, the important role of the POA in both males and females, and translational sex research from animals to humans. He and Vlasta never had children. He was the last person to bear the family name Madlafousek in Czechia, and probably in the world. But he lives on through his colleagues and intellectual offspring in Czechia and Slovakia, and in those throughout the world who discover the treasure of his contributions.

## Supplementary Information

Below is the link to the electronic supplementary material.

Supplementary file1 (DOCX 32 kb)

Supplementary file2 (DOCX 6325 kb)
