# Supplementary material for: Not To Be Forgotten: Jaroslav Madlafousek’s Important Contributions to Sex Research
Source: Arch Sex Behav. 2025 Aug 18;54(8):3071–8. doi: 10.1007/s10508-025-03218-y (PMC12484094; doi:10.1007/s10508-025-03218-y)
Supplement: Supplementary file 1 — Supplementary file1 (DOCX 32 kb) [file 10508_2025_3218_MOESM1_ESM.docx]

**Bibliography of Jaroslav Madlafousek**

Gerova, M., & Madlafousek, J. (1956). Impedance plethysmography as a method of measuring

the blood flow through an extremity. *Physiologia Bohemoslovaca, 5*(1)*,* 114-120.

Gerova, M., & Madlafousek, J. (1956). Impedanční pletysmografie jako metoda měření cirkulace

v končetinách [Impedant plethysmography as a method of measurement of circulation of

the extremity]. *Československá Fysiologie, 5*(1)*,* 100-106.

Madlafousek, J. (1957). Orientace reakcí jako počáteční složka adaptivního vybavení organismu

[Orientation reactions as an initial component of the organism's adaptive equipment].

*Ceskoslovenská Psychologie 1(1),* 39-44.

Brod, J., Fencl, V., Hejl, Z., Jirka, J., & Madlafousek, J. (1958). Změny prokrvení svalů a kůže

v předloktí během emočního stresu [Changes of muscle and skin blood supply in the

forearm during emotional stress]. *Československá Fysiologie, 7*(5)*,* 437-438.

Fencl, V., Hejl, Z., Jirka, J., Madlafousek, J., & Brod, J. (1959). Changes of blood flow in

forearm muscle and skin during an acute emotional stress (mental arithmetic). *Clinical*

*Science, 18,* 491-498.

Madlafousek, J. (1961). Příspěvek tzv. pití ve skupině k rozvoji alkoholismu u jednotlivce [The

contribution of the so-called drinking party to the development of alcoholism in an

individual]. *Zpravodaj Ustředního Protialkoholního Sboru MZd, 3*(1)*,* 1-25.

Madlafousek, J., & Freund, K. (1962). Skutečné zvířecí modely [Real animal models].

*Ceskoslovenská Psychiatrie, 58*(1)*,* 70-71.

Madlafousek, J. (1962). Fylogeneze a psychologie [Phylogeny and psychology]. Učební texty

vysokých škol. FF UK.

Madlafousek, J., & Freund, K. (1964). Experimentální psychopatologie [Experimental

psychopathology]. *Zprávy VUPs, 9,* 1.

Madlafousek, J. (1964). Vegetativní složky duševních činností [Vegetative components of

mental activities]. Kandidátská práce, Universita Karlova.

Madlafousek, J. (1964). Studium motivace pomocí elektrické stimulace mozku [Studying

motivation using electrical brain stimulation]. *Ceskoslovenská*

*Fysiologie, 13*(4)*,* 309-315.

Irmiš, F., Madlafousek, J., Hliňák, Z. (1968). Změny aktivity EEG v hipokampu během

páření u samců potkanů [Changes in hippocampal EEG activity during copulation in male

rats]. *Ceskoslovenská Fysiologie, 17*(3)*,* 224-225.

Hliňák, Z., & Madlafousek, J. (1968). Analýza sexuálního chování u samic potkanů a otázka

kritéria intenzity motivace [Analysis of sexual behavior in female rat and a question

of criterion of motivation intensity]. *Československá Psychologie, 5,* 516-519.

Madlafousek, J., Hliňák, Z., & Freund, K. (1968). Experimentální psychopatologie

[Experimental psychopathology]. *Zprávy VUPs, 13,* 24-25.

Madlafousek, J., & Grofová, I. (1968). Etologické pojmy autostimulačního chování [Ethological

concepts of autostimulatory behavior]. *Ceskoslovenská Psychologie, 12*(5)*,* 504-506.

Hliňák, Z., & Madlafousek, J. (1969). A quantitative study of the synergistic action of oestradiol

and progesterone in inducing the oestrous behaviour of the ovariectomized rat.

*Physiologia Bohemoslovaca, 18,* 485-486.

Hliňák, Z., & Madlafousek, J. (1969). A shortening effect of progesterone on the duration of the

oestrous behaviour in the female rat. *Physiologia Bohemoslovaca, 18,* 340.

Irmiš, F., Madlafousek, J., & Hliňák, Z. (1970). Hippocampal electrical activity in course of

sexual behavior of male rats. *Physiologia Bohemoslovaca, 19*(1)*,* 83-87.

Hliňák, Z., & Madlafousek, J. (1970). První heterosexuální zkušenosti samce krysy [First

heterosexual experiences of a male rat]. *Československá Psychologie, 14,* 278-279.

Madlafousek, J., Freund, K., & Grofová, I. (1970). Variables determining the effect of

electrostimulation in the lateral preoptic area on the sexual behavior of male rats.

*Journal of Comparative and Physiological Psychology, 72*(1)*,* 28-44. doi:

10.1037/h0029307.

Hliňák, Z., Madlafousek, J., Gutmann, E., & Hanzlíková, V, (1971). Copulatory activity of male

rats after denervation of the levator ani muscle. *Physiologia Bohemoslovaca, 20,* 374-

375.

Hliňák, Z., & Madlafousek, J. (1971). The effectiveness of estradiol and progesterone in

inducing estrous behavior of rats ovariectomized at different ages. *Physiologia*

*Bohemoslovaca, 20,* 64-65.

Hliňák, Z., & Madlafousek, J. (1971). The dependence of sexual behavior of inexperienced

males on the precopulatory behavior of female albino rats. *Proceedings of the*

*Czechoslovak Physiological Society, 21,* 83.

Madlafousek, J., Hliňák, Z., & Parízek, J. (1971). Sexual behaviour of male rats sterilized by

cadmium. *Journal of Reproduction and Fertility, 26*(2)*,* 189-196. doi:

10.1530/jrf.0.0260189.

Madlafousek, J., & Hliňák, Z. (1971). První kopulace dospělých samců mění jejich závislost na

předkopulačním chování samic (u potkanů). Je to bod učení? [The first copulations of

adult males change their dependence on the female precopulatory behavior (in rats). Is

this a point of learning?]. *Československá Psychologie, 15,* 1-11.

Kolářský, A., & Madlafousek, J. (1972). Female behavior and sexual arousal in heterosexual

male deviant offenders. *Journal of Nervous and Mental Disorders, 155*(2)*,* 110-118. doi:

10.1097/00005053-197208000-00005.

Hliňák, Z., & Madlafousek, J. (1972). The dependence of sexual behavior of inexperienced

males on the precopulatory behavior of females in albino rat. *Physiologia*

*Bohemoslovaca, 21,* 83-84.

Hliňák, Z., & Madlafousek, J. (1972). Positive and negative effects of progesterone on the

precopulatory behavior of ovariectomized rats. *Activitas Nervosa Superior (Praha),*

*14*(3)*,* 170-171.

Madlafousek, J., and Hliňák, Z. (1972). Analysis of factors determining the appetitive and

aversive phase of sexual behavior in the female rat. *Physiologia Bohemoslovaca, 21,*

416-417.

Hliňák, Z., & Madlafousek, J. (1974). Sexual arousal in mouse-killing male rats. *Physiologia*

*Bohemoslovaca, 23,* 146-147.

Kolářský, A., Madlafousek, J., Hliňák, Z., & Novotná, V. (1975). Podivná žena jako objekt

deviantního muže a biologie fází sexuálního chování [Strange woman as object of

deviant male and biology of phases of sexual behavior]. *Československá Psychiatrie,*

*71*(5)*,* 291-294.

Madlafousek, J., Hliňák, Z., & Beran, J. (1976). Decline of sexual behavior in castrated male

rats: effects of female precopulatory behavior. *Hormones and Behavior, 7*(2)*,* 245-252.

doi: 10.1016/0018-506x(76)90051-9.

Madlafousek, J. (1976). Autoregulated intracranial stimulation (self-stimulation) as an induction

of a cycle of motivational states. *Activitas Nervosa Superior (Praha), 18*(1-2)*,* 97-101.

Kolářský, A., & Madlafousek J. (1977). Variability of stimulus effect in the course of

phallometric testing. *Archives of Sexual Behavior, 6*(2)*,* 135-141. doi:

10.1007/BF01541705.

Hliňák, Z., & Madlafousek J. (1977). Female precopulatory behaviour as a determinant of sexual

activity in male rats [proceedings]. *Activitas Nervosa Superior (Praha). 19*(3)*,* 242-243.

Madlafousek, J., & Martínek, Z. (1977). Psychologie zvířat [Psychology of animals]. In: Naučný

slovník zemědělský, Vol. 7. Praha SZN, 550-560.

Madlafousek, J., & Hliňák Z. (1977). Sexual behaviour of the female laboratory rat:

Inventory, patterning, and measurement. Behaviour, *63*(3/4)*,* 129-174.

<http://www.jstor.org/stable/4533852>

Madlafousek, J., & Hliňák, Z. (1978). Chování jako integrovaný proces: metodologické,

metodické a technické problémy [Behavior as an integrated process: Methodological,

methodical, and technical problems]. *Zprávy Výzkumného ústavu psychiatrie*, *54,*

1-42.

Kolářský, A., Madlafousek, J., & Novotná, V. (1978). Stimuli eliciting sexual arousal in males

who offend adult women: An experimental study. *Archives of Sexual Behavior, 7*(2)*,* 79-

87*.* doi: 10.1007/BF01542057.

Hliňák, Z., & Madlafousek, J. (1978). Factors influencing the duration of copulatory posture in

female laboratory rat [proceedings]. *Activitas Nervosa Superior (Praha), 20*(2)*,* 115.

Madlafousek, J., Hliňák, Z. (1978). Od předkopulačního chování po kopulační chování (u samců

laboratorních potkanů): popis, analýza, hypotézy [From pre-copulatory to copulatory

behavior (in the male laboratory rat): Description, analysis, hypotheses]. *Ceskoslovenská*

*etologická společnost, 5.*

Madlafousek, J. (1978). Tvorba behaviorálních kategorií [Formation of behavioral categories].

*Zprávy VUPs, 54,* 15-18.

Madlafousek, J. (1978). Koncept a problém integrovaného chování [The concept and problem of

integrated behavior]. *Zprávy VUPs, 54,* 7-8.

Madlafousek, J. (1978). Intenzivní aspekty pozorovaného chování [Intense aspects of observed

behavior]. *Zprávy VUPs,* 54, 22-24.

Madlafousek, J. (1978). Co je to etologie? [What is ethology?]. *Biologické Listy 43*(1)*,* 56-58.

Hliňák, Z., Madlafousek, J., & Mohapelová, A. (1979). Initiation of copulatory behavior in

castrated male rats injected with critically adjusted doses of testosterone. *Hormones*

*and Behavior, 13*(1)*,* 9-20. doi: 10.1016/0018-506x(79)90031-x.

Hliňák, Z., & Madlafousek, J. (1979). Copulatory behaviour of castrated male rats injected with

suboptimal testosterone doses [proceedings]. *Activitas Nervosa Superior (Praha), 21*(1)*,*

52-53.

Madladfousek, J. (1979). Problémy učení v motivačních systémech [Problems of learning in

motivational systems]. *Ceskoslovenská psychologie 23*(6)*,* 529-538.

Madlafousek, J. (1979). Zpráva o plnění národního programu základního výzkumu v psychiatrii

[A report on the fulfillment of the national program of basic research in psychiatry].

*Československá Psychiatrie, 76*(2)*,* 280-284.

Kolářský, A., & Madlafousek, J. (1980). Vzrušuje exhibicionisty strach nebo vztek žen? [Are

exhibitionists excited by female fear or anger?]. *Casopís Lékar̆ů C̆eských, 119*(17-18)*,*

497-500.

Hliňák, Z., & Madlafousek, J. (1980). Sniffing and other exploratory behaviors in relation

to copulatory readiness in male rats. *Activitas Nervosa Superior (Praha), 22*(2)*,* 89-90.

Hliňák, Z., & Madlafousek, J. (1980). The critical state of copulatory readiness in castrated

male rats induced through testostosterone filled silastic capsules. *Physiologia*

*Bohemoslovaca, 29*(5)*,* 442.

Benešová, O., Madlafousek, J., & Hliňák, Z. (1980). Activity and defecation rate in

“open-field” as related to the copulatory readiness in male rats. *Activitas Nervosa*

*Superior (Praha), 22*(2)*,* 90-91

Madlafousek, J., & Hliňák, Z. (1980). On the problem of specificity in brain stimulation

experiments. *Physiologia Bohemoslovaca, 29*(5)*,* 455.

Madlafousek, J., & Hliňák, Z. (1980). On the sexual dyad in laboratory rat. *Activitas Nervosa*

*Superior (Praha), 22*(4)*,* 289-296.

Madlafousek, J., & Hliňák, Z. (1980). Normální a patologické chování samců laboratorních

potkanů během narušeného přechodu od předkopulačního chování ke kopulačnímu

chování [Normal and pathological behavior of male laboratory rats during the impeded

transition from the precopulatory to copulatory behavior]. *Activitas Nervosa Superior*

*(Praha), 22*(1)*,* 24-25

Madlafousek, J. (1980). Zpráva o výzkumných a publikačních činnostech VÚP za období

1976-1980 [Report on research and publication activities of VÚPs for the period

1976-1980.] *Pražský psychiatrický výzkumný ústav* (Pp 1-91).

Hliňák, Z., Matoušek, J., & Madlafousek, J. (1981). The effect of bull seminal ribonuclease on

reproductive organs and sexual behaviour in male rats. *Physiologia Bohemoslovaca,*

*30*(6)*,* 539-542.

Hliňák, Z., & Madlafousek, J. (1981). Estradiol treatment and precopulatory behavior in

ovariectomized female rats. *Physiology & Behavior, 26*(2)*,* 171-176. doi: 10.1016/0031-

9384(81)90006-8.

Madlafousek, J., Žantovský, M., Hliňák, Z., & Kolářský, A. (1981). Sexuální chování jako

komunikační proces, kterým se realizuje systém částečných motivačních stavů [Sexual

behavior as a communicative process by which a system of partial motivational states is

realized]. *Československá Psychiatrie, 77*(6)*,* 377-384.

Hliňák, Z., Madlafousek, J., & Krejčí, I. (1982). The influence of lisuride on sexual behavior

in male rats. *Activitas Nervosa Superior (Praha), 24*(4)*,* 204-205.

Hliňák, Z., & Madlafousek, J. (1982). Initiation of copulatory behaviour in castrated male rats

implanted with very small testosterone-filled silastic capsules. *Endokrinologie, 79*(1)*,* 35-

43.

Hliňák, Z., & Madlafousek, J. (1982). Under what conditions does laboratory male rat initiate

copulatory behaviour with passively receptive female. *Activitas Nervosa Superior*

*(Praha), 24*(1)*,* 9-13.

Madlafousek, J. & Hliňák, Z. (1983). Importance of female’s precopulatory behaviour for the

primary initiation of male’s copulatory behaviour in the laboratory rat. *Behaviour, 86*(3-

4)*,* 237-248.

Kolářský, A., & Madlafousek, J. (1983). The inverse role of preparatory erotic stimulation in

exhibitionists: Phallometric studies. *Archives of Sexual Behavior, 12*(2)*,* 123-148. doi:

10.1007/BF01541557

Hliňák, Z., & Madlafousek, J. (1983). Estradiol plus progesterone treatment and precopulatory

behavior in ovariectomized female rats. *Physiology & Behavior, 30*(2)*,* 221-227. doi:

10.1016/0031-9384(83)90009-4.

Hliňák, Z., & Madlafousek, J. (1983). Factors influencing the initiation of copulatory behavior of

male laboratory rats with a passively receptive female. *Activitas Nervosa Superior*

*(Praha), 25*(3)*,* 181-182.

Kolářský, A., Madlafousek, J., & Zvĕrina, J. (1984). Zlepšení sexuálního partnerství

exhibicionistů [Improvement of the sexual parternership of exhibitionists].

*Československá Psychiatrie, 80*(5)*,* 303-307.

Hliňák, Z., & Madlafousek, J. (1984). Precopulatory behavior following testosterone

treatment in castrated male rats. *Activitas Nervosa Superior (Praha), 26*(3)*,* 224-225.

Madlafousek, J., Kolářský, A., & Zvĕrina, J. (1985). Penile volume response to female emotional

behavior in men, committing forcible sexual acts. *Activitas Nervosa Superior (Praha),*

*27*(2)*,* 151-152.

Madlafousek, J. (1985). Závěrečná zpráva o plnění státního plánu základního výzkumu [Final

report on the implementation of the State Basic Research Plan]. Pražský psychiatrický

výzkumný ústav

Madlafousek, J., & Freund, K. (1986). Experimentální neuropsychopatologie. (Modely na

zvířatech) [Experimental neuropsychopathology. (Models in animals)]. *VUPs Reports,*

*60,* 19-20.

Hliňák, Z., Madlafousek, J., & Špinka, M. (1987). Transition from precopulatory to copulatory

behaviour in male rats with lesions in medial preoptic area: dependence on

precopulatory pattern of female. *Activitas Nervosa Superior (Praha), 29*(4)*,* 257-263.

Hliňák, Z., Špinka, M., Madlafousek, J., & Semerád, F. (1988). Role of the medial preoptic area

in sexual behaviour of the male rat: a study using repeated cycloheximide infusions.

*Physiologia Bohemoslovaca, 37*(5)*,* 432-442.

Bartoš, L., Rödl, P., & Madlafousek, J. (1988). Induction of copulatory behavior in castrated

female and male Arctic foxes. *Hormones and Behavior, 22*(4)*,* 467-473. doi:

10.1016/0018-506x(88)90051-7.

Losos, S., & Madlafousek, J. (1992). Classification of fallow deer groups according to size

under deer park conditions. In: Brown, R. D. (ed.), *The biology of deer* (P. 88). Springer.

https://doi.org/10.1007/978-1-4612-2782-3_23

Madlafousek, J. (1993). Erotologie? [Erotology?]. *Newsletter of the Czech and Slovak*

*Ethological Society, 8,* 6-9.

Bartoš, L., & Madlafousek, J. (1994). Infanticide in a seasonal breeder: the case of red deer.

*Animal Behaviour, 47,* 217-219.

Madlafousek, J. (1994). Láska k bližnímu v pleistocénu a dnes [Love of neighbour in Pleistocene

and today]. *Československá Psychologie, 38*(1)*,* 53-65.

Illmann, G. & Madlafousek, J. (1995). Occurrence and characteristics of unsuccessful nursings

in minipigs during the first week of life. *Applied Animal Behavior Science, 44,* 9-18.

Madlafousek, J. (1995). O vývoji pojmu „funkce“ v evoluční biologii [On the development of

the concept of "function" in evolutionary biology]. *Newsletter of the Czech and Slovak*

*Ethological Society, 10,* 5-8.

Madlafousek, J (1996). Kurt Freund (1914-1996). *Československá Psychologie, 40*(6)*,* 555-

556.

Madlafousek, J. (1997). Mozek není univerzální superpočítač [The brain is not a universal

supercomputer]. *PROPS 3(1),* 10-11.

Madlafousek, J. (1997). Dospívání chlapců a dívek bez otců: psychosociální deprivace

nebo alternativní adaptace? [Adolescence of boys and girls without fathers: psychosocial

deprivation or alternative adaptation?]. *Československá Psychologie, 41*(5)*,* 451-454.

Madlafousek J. (1997). Fenomén učení z pohledu současné etologie a evoluční psychologie [The

phenomenon of learning in the perspective of contemporary ethology and evolutionary

psychology]. *Newsletter of the Czech and Slovak Ethological Society, 10,* 5-8.

Madlafousek, J. (1999). Dawkinsova kniha „The Common Gene“ (Společný gen) pro

behaviorální vědy [Dawkins' "The Common Gene" for behavioral sciences]. *Psychiatrie,*

*3*(1), 74-77.

Madlafousek, J. (2001): Rozhovor s PhDr. Jaroslavem Madlafouskem (2000–2001) [Interview

with PhDr. Jaroslav Madlafousek (2000-2001)]. In: Baštecká, B., & Goldmann, P.

*Základy klinické psychologie* (Pp 140-148). Portál.

Madlafousek, J. (2002). Evoluční psychologie [Evolutionary psychology]. In: C. Höschl, J.

Libiger, J. Švestka (Eds.), *Psychiatrie* (Pp. 212-216). Tigis.

Madlafousek, J. (2002). Evoluční psychologie – příklad sjednocující psychologie [Evolutionary

psychology – An example of unifying psychology]. *Psychologie Dnes, 8*(2)*,* 22-23.

Madlafousek, J., & Hučín, J. (2002). Situace současného člověka se změnila [The situation of

contemporary man has changed]. *Psychologie Dnes, 8*(5)*,* 13.

Madlafousek, J. (2005). Pro budoucí rodiče [For parents in the making]. In: *Manere in montibus;*

*Proceedings of Václav Brichacek on the occasion of his life jubilee* (Pp. 135-140). UK

FHS

Madlafousek J. Člověk, jeho rodina a společnost [The human child, his family and society].

Unfinished book.
