# Supplementary material for: Not To Be Forgotten: Jaroslav Madlafousek’s Important Contributions to Sex Research
Source: Arch Sex Behav. 2025 Aug 18;54(8):3071–8. doi: 10.1007/s10508-025-03218-y (PMC12484094; doi:10.1007/s10508-025-03218-y)
Supplement: Supplementary file 2 — Supplementary file2 (DOCX 6325 kb) [file 10508_2025_3218_MOESM2_ESM.docx]

**Supplemental Photos and figures**

**
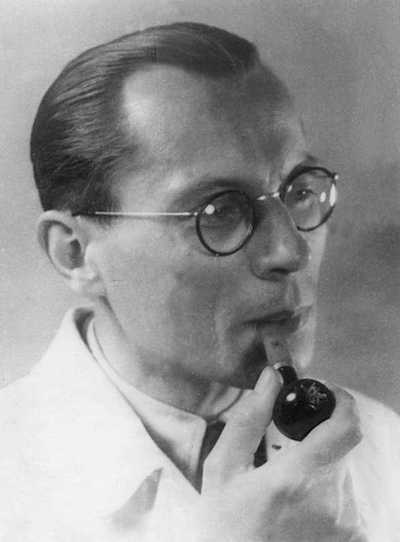
**

**Figure 1:** Josef Stavěl, Ph.D. (1901-1986), Madlafousek’s undergraduate and graduate mentor in psychology at Charles University in Prague.


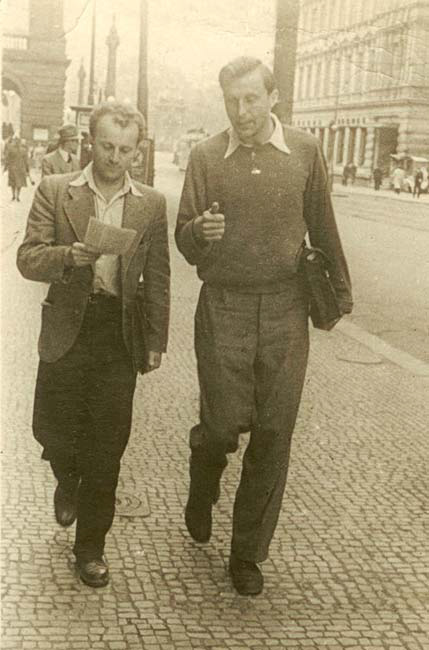


**Figure 2:** Madlafousek in 1947 with fellow student Josef Lát near the Faculty of Arts building outside the Rudolfinum concert hall and gallery in Prague. Note the archway behind them.

**
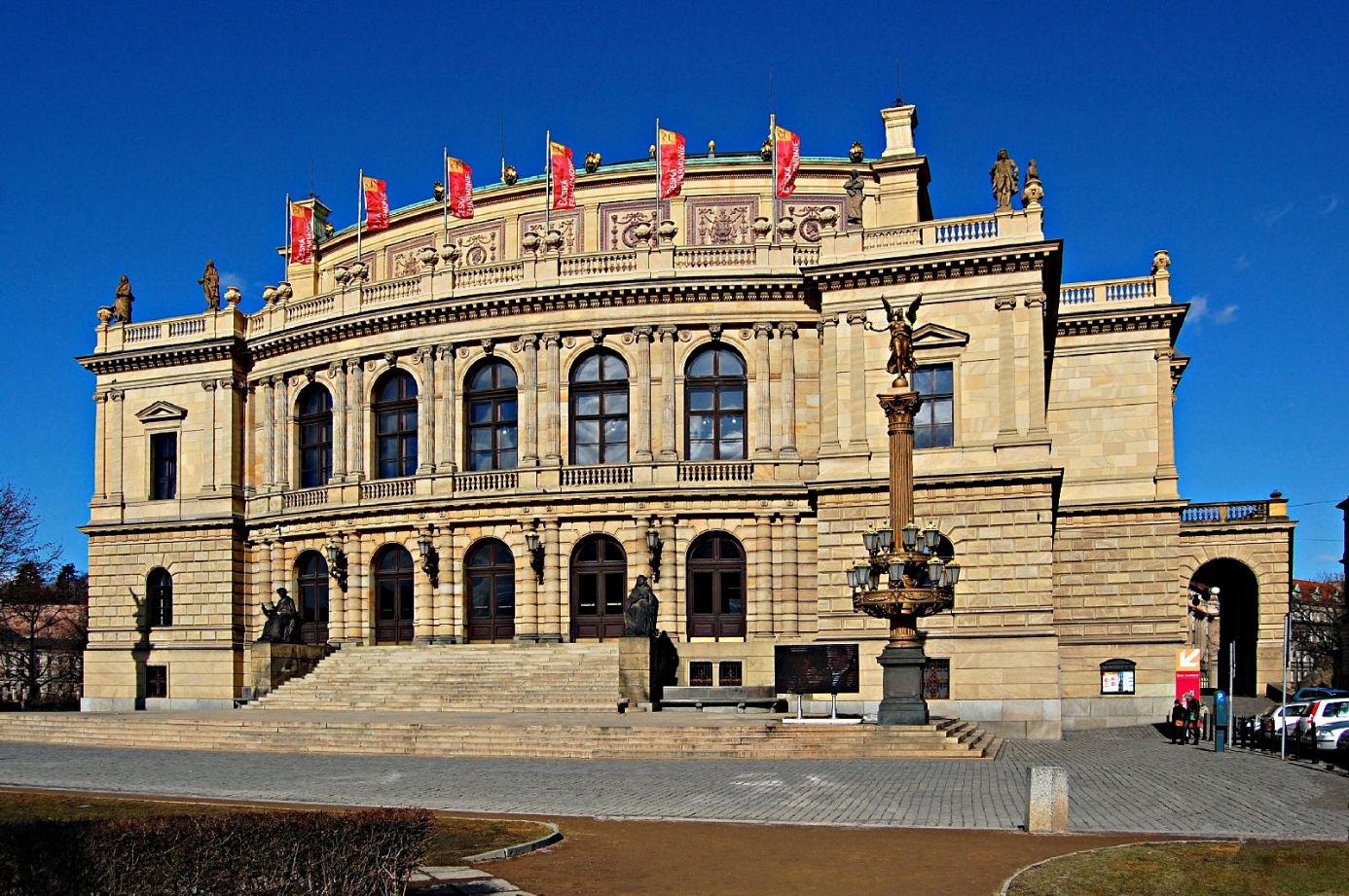
**

**Figure 3:** The Rudolfinum today, in front of what is now known as Jan Palach Square, in honor of the 20-year old student who immolated himself on 19 January 1969 as a political protest against the Soviet invasion and end of the Prague Spring. Note the archway to the right.


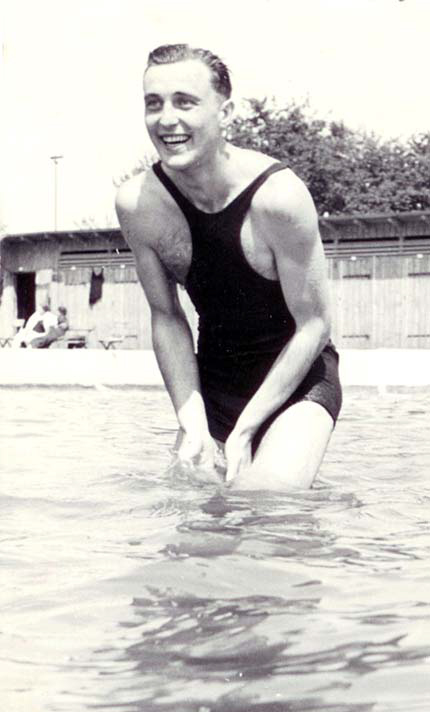


**Figure 4:** Madlafousek, age 27, in the summer of 1949 before his doctoral defense.

**
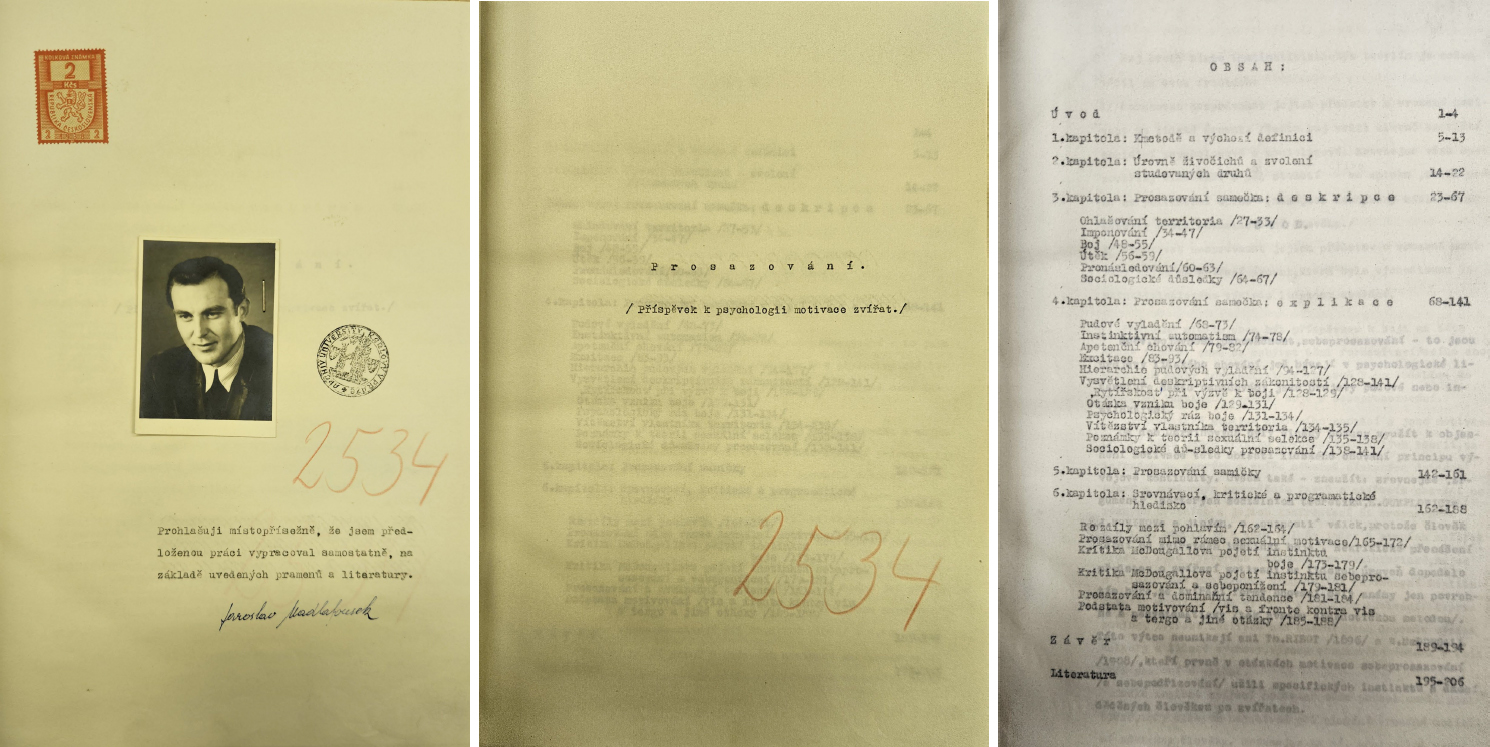
Figure 5:** Madlafousek’s PhD thesis. Left to right: Title pages and Table of Contents (Czech: *Obsah*).

**
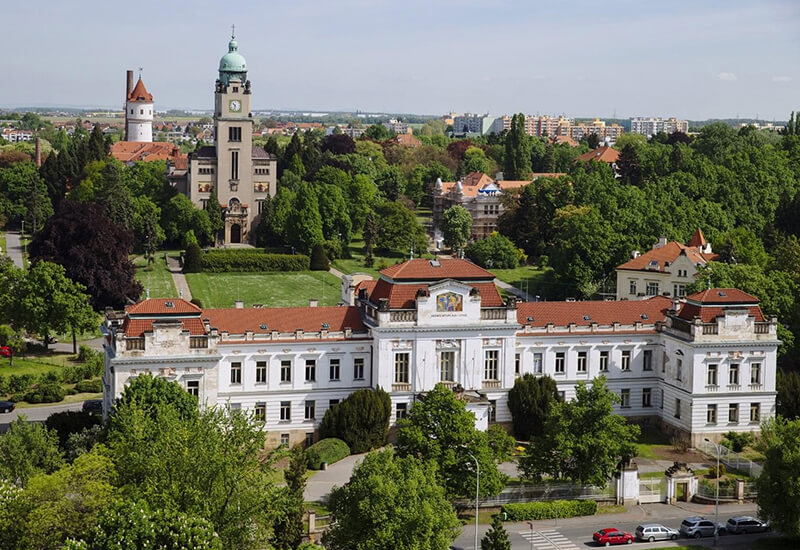
Figure 6:** Campus of the Prague Psychiatric Center at Bohnice.


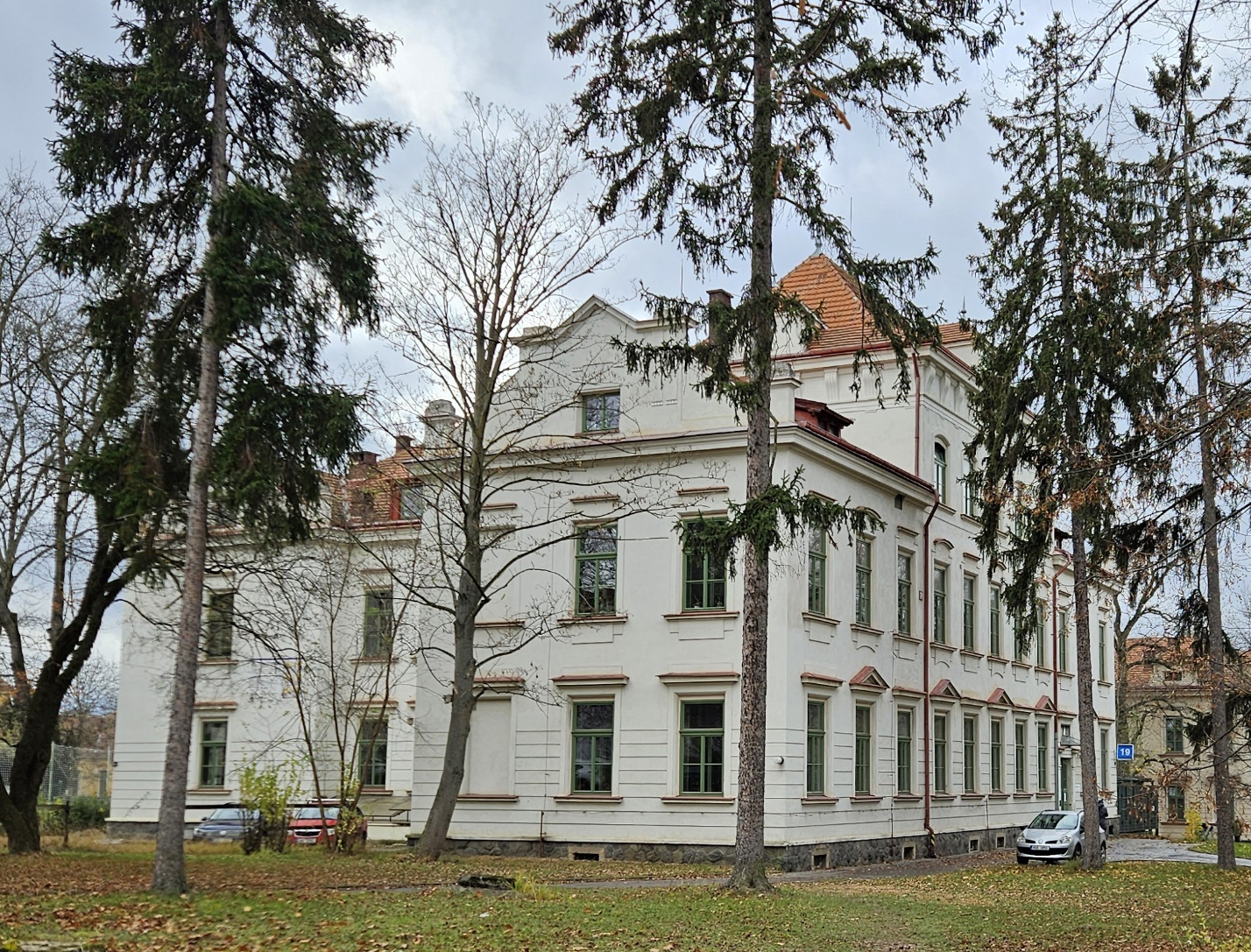


**Figure 7:** Pavilion 19 where Madlafousek had his rat laboratory in the basement.


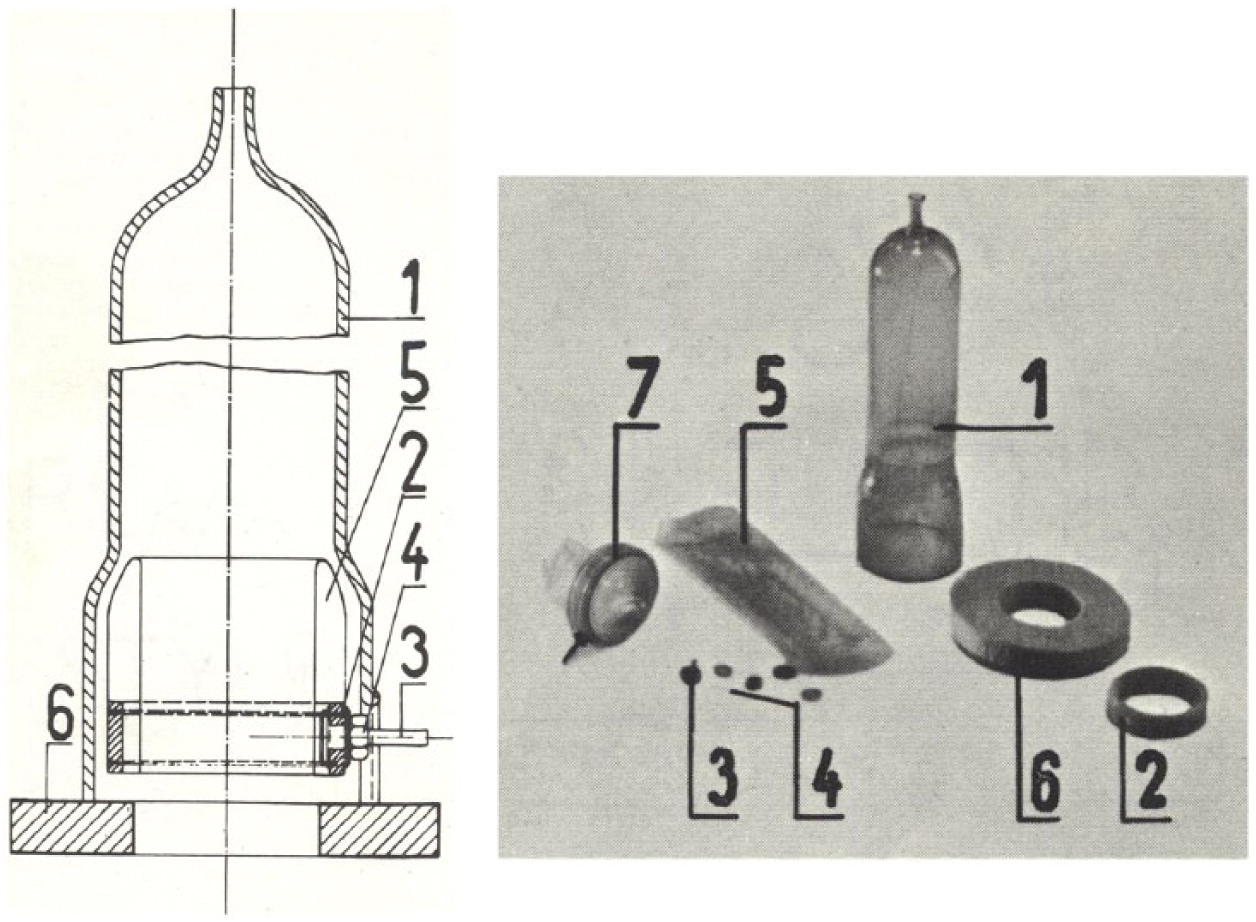


**Figure 8:** Volumetric Air Chamber (VAC) device invented by Kurt Freund for volumetric penile plethysmography to assess penile erection objectively. This entailed fitting a sealed tube over the penis so that changes in length and diameter could be detected as air displacement from the tube that was recorded by a pen on a rotating drum. In the late 1950s and early 1960s, a substantial proportion of men tried to avoid compulsory military service in Czechoslovakia by feigning homosexuality. Freund and Madlafousek showed men a series of erotic still pictures of nude men and women, and from the erectile responses, they could weed out the men that were truly homosexual from those that were not. Freund brought the device to North America when he left Czechoslovakia. Photo from Freund et al. (1965). Madlafousek continued to use it as both a research and diagnostic tool at the Prague Psychiatric Center along with impedance plethysmography that he perfected.


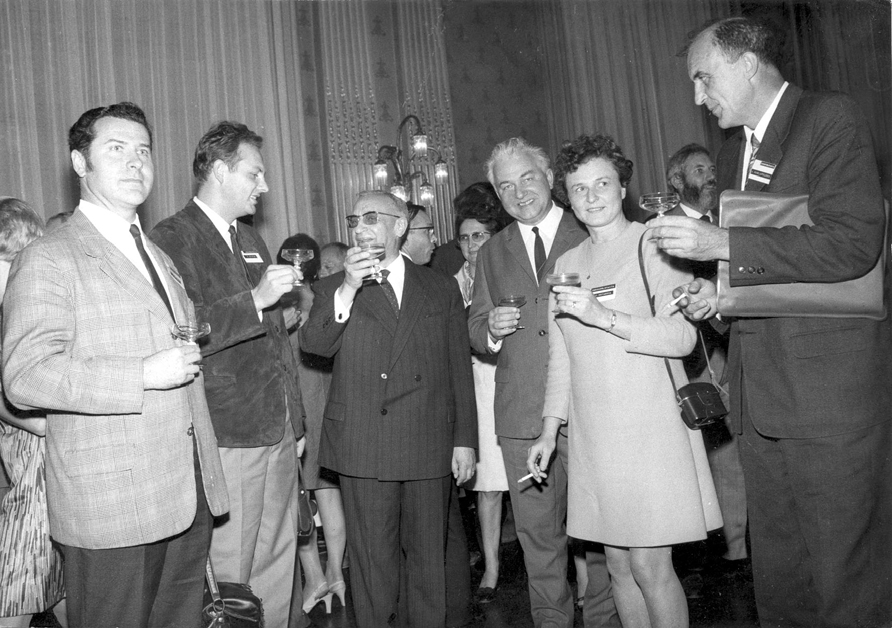


**Figure 9:** Madlafousek (extreme right) in 1969, age 47, at the XI International Ethological Conference in Rennes, France.


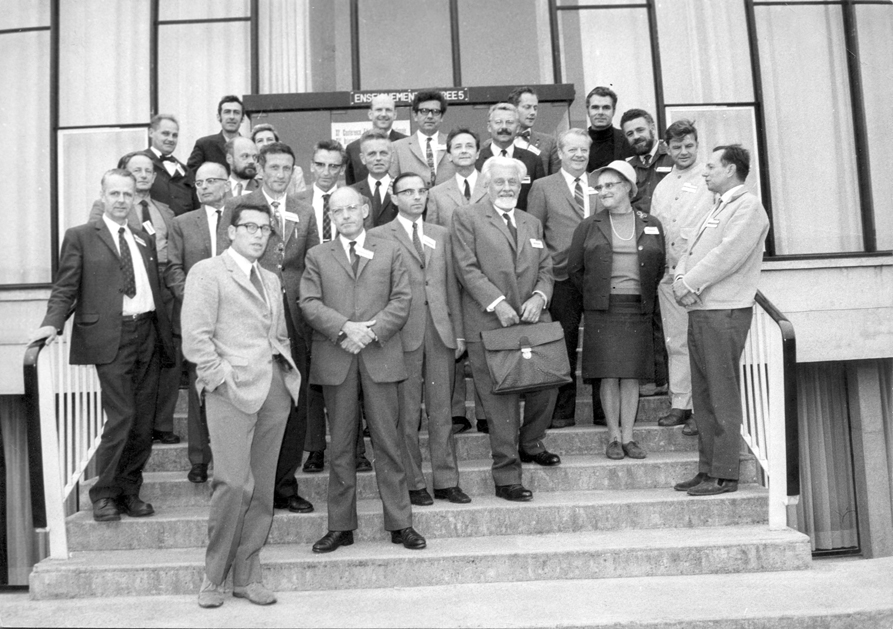


**Figure 10:** XI International Ethological Conference speakers, including Konrad Lorenz (right of center). Photo by Madlafousek.


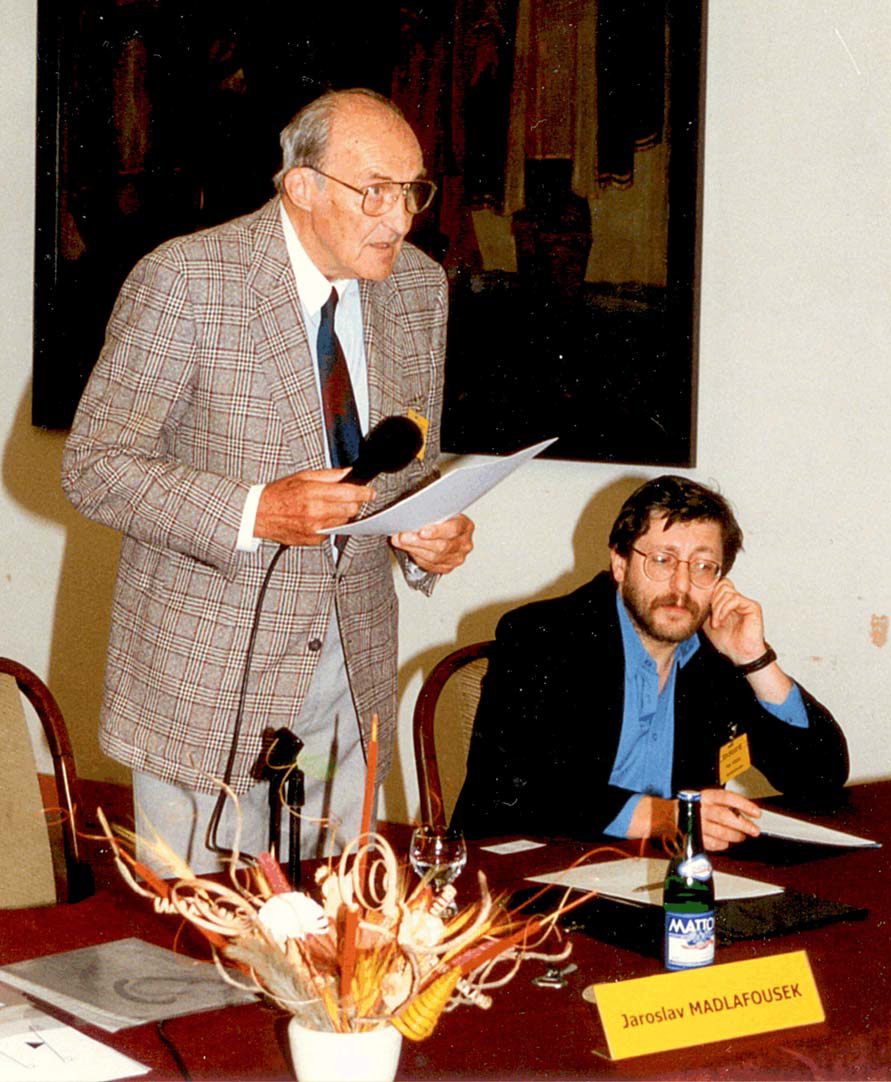


**Figure 11:** Madlafousek in 1998 (age 76) at Prague Brain Day with Prof. Petr Weiss.


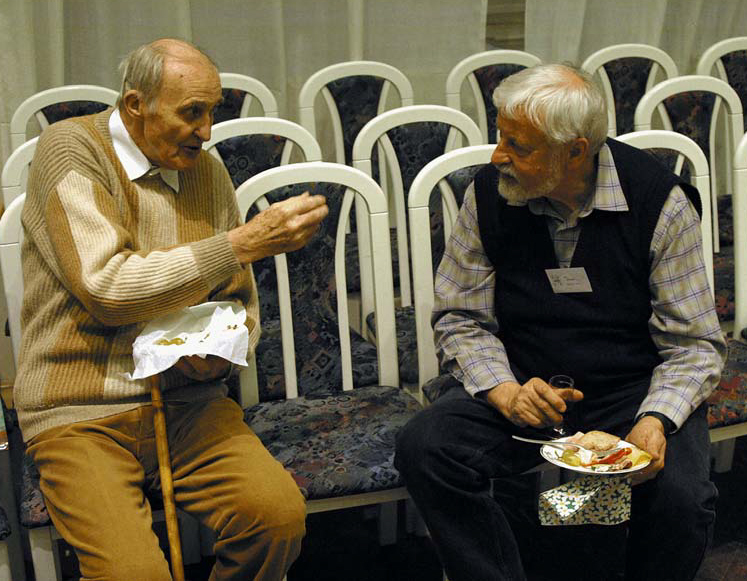


**Figure 12:** Madlafousek during a celebration of his contributions and upcoming birthday at the XXX Czech and Slovak Ethological Conference in Jičín in April 2003 (with prof. Jan Žďárek).


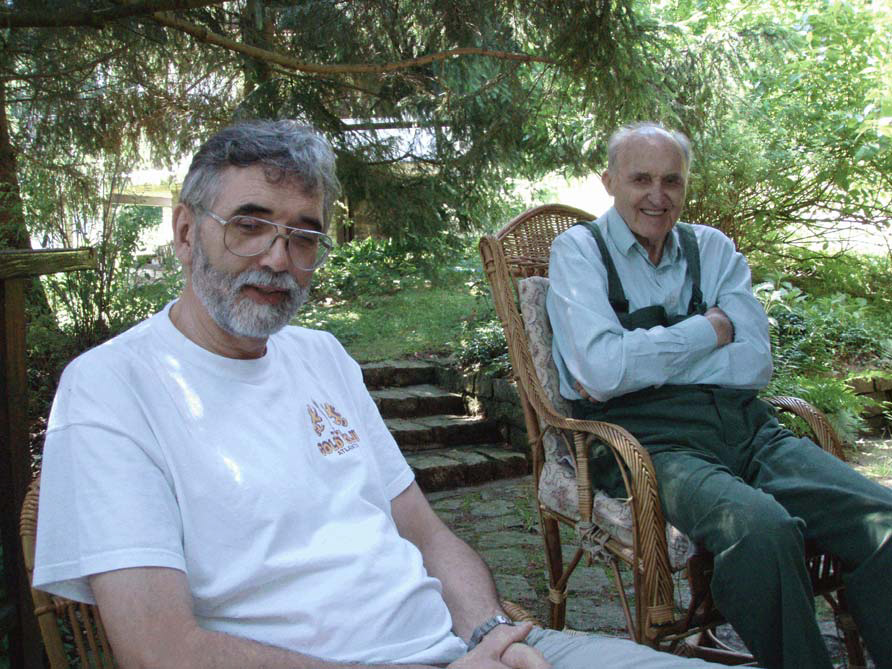


**Figure 13:** On of the last photos of Madlafousek taken at the cottage in Orlík nad Vltavou (with Luděk Bartoš on the left)


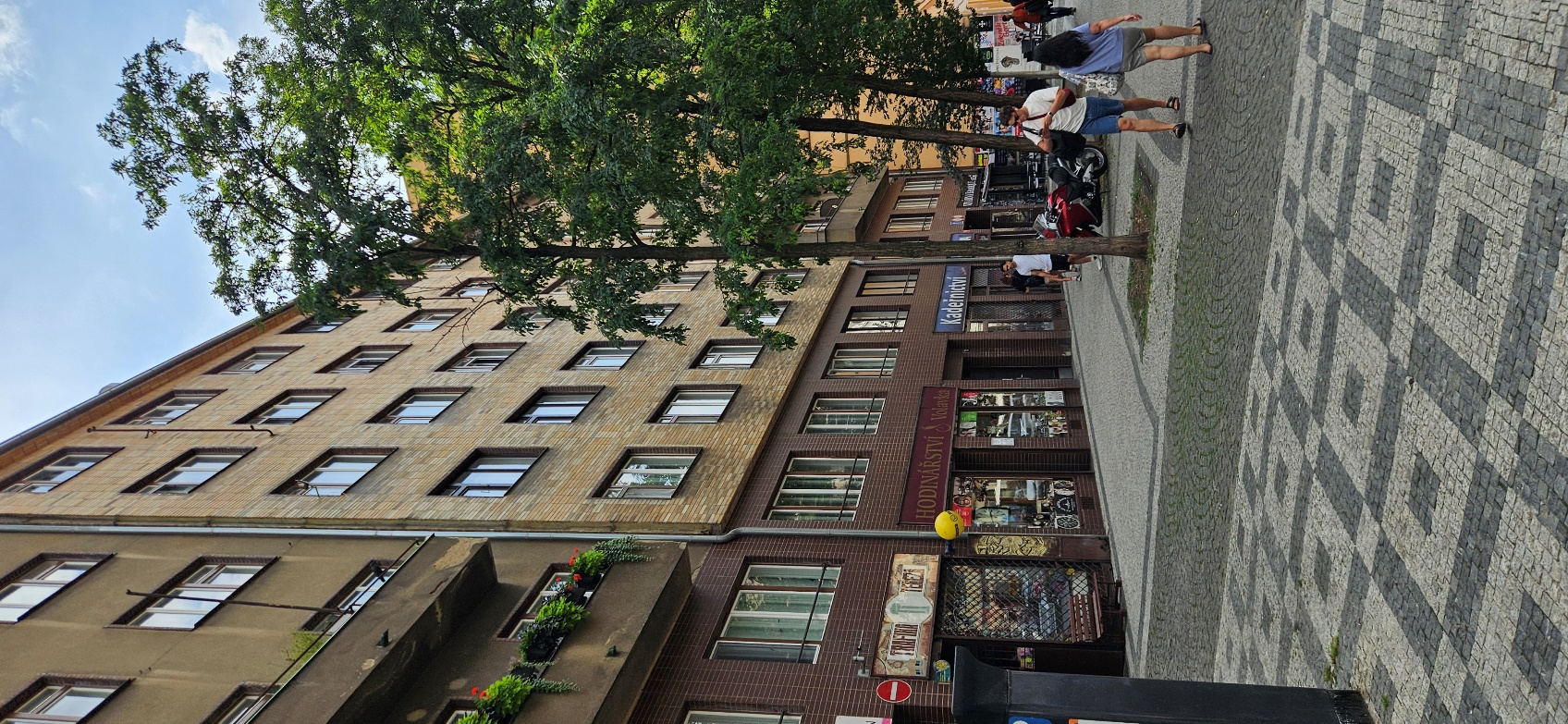


**Figure 14:** Jarka’s flat at Sokolovská 72, Praha 8 – Karlín, where he and Vlasta lived from the 1950s.


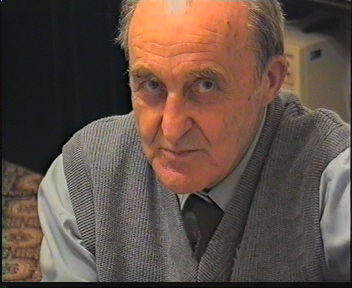


**Figure 15:** Madlafousek, summer of 1994 (Age 72). Still from a video taken during a seminar in Uhříněves.
